# Spatial epidemiology of hemorrhagic disease in Illinois wild white-tailed deer

**DOI:** 10.1038/s41598-022-10694-y

**Published:** 2022-04-27

**Authors:** Sheena J. Dorak, Csaba Varga, Mark G. Ruder, Peg Gronemeyer, Nelda A. Rivera, Douglas R. Dufford, Daniel J. Skinner, Alfred L. Roca, Jan Novakofski, Nohra E. Mateus-Pinilla

**Affiliations:** 1 Illinois Natural History Survey – Prairie Research Institute, University of Illinois Urbana-Champaign, 1816 S. Oak Street, Champaign, IL 61820 USA; 2 Department of Pathobiology, University of Illinois Urbana-Champaign, 2001 South Lincoln Avenue, Urbana, IL 61802 USA; 3 Southeastern Cooperative Wildlife Disease Study, College of Veterinary Medicine, University of Georgia, Athens, GA 30602 USA; 4 Illinois Department of Natural Resources, One Natural Resources Way, Springfield, IL 62702 USA; 5 Department of Animal Sciences, University of Illinois Urbana-Champaign, 1207 West Gregory Drive, Urbana, IL 61801 USA

**Keywords:** Viral epidemiology, Ecological epidemiology, Infectious diseases

## Abstract

Epizootic hemorrhagic disease (EHD) and bluetongue (BT) are vector-borne viral diseases that affect wild and domestic ruminants. Clinical signs of EHD and BT are similar; thus, the syndrome is referred to as hemorrhagic disease (HD). Syndromic surveillance and virus detection in North America reveal a northern expansion of HD. High mortalities at northern latitudes suggest recent incursions of HD viruses into northern geographic areas. We evaluated the occurrence of HD in wild Illinois white-tailed deer from 1982 to 2019. Our retrospective space–time analysis identified high-rate clusters of HD cases from 2006 to 2019. The pattern of northward expansion indicates changes in virus-host-vector interactions. Serological evidence from harvested deer revealed prior infection with BTV. However, BTV was not detected from virus isolation in dead deer sampled during outbreaks. Our findings suggest the value of capturing the precise geographic location of outbreaks, the importance of virus isolation to confirm the cause of an outbreak, and the importance of expanding HD surveillance to hunter-harvested wild white-tailed deer. Similarly, it assists in predicting future outbreaks, allowing for targeted disease and vector surveillance, helping wildlife agencies communicate with the public the cause of mortality events and viral hemorrhagic disease outcomes at local and regional scales.

## Introduction

Hemorrhagic disease (HD) is an acute and often fatal vector-borne disease that affects white-tailed deer (WTD; *Odocoileus virginianus*) caused by either epizootic hemorrhagic disease virus (EHDV) or bluetongue virus (BTV). In the United States of America (USA), EHDV was first isolated in 1955 following an outbreak in WTD in New Jersey^1^, and BTV was implicated in the etiology of HD in 1966. However, the viruses may have been present in the USA long before this, as anecdotal reports consistent with HD in deer date back to the late 1800s^2,3^. In the 1950s, deer die-offs were reported at more northern latitudes, including the states of New Jersey, Michigan, North Dakota, and Washington in the USA, and Canada in the 1960s^2,4^. Although controlled studies targeting EHDV and BTV separately exist, few examine outbreaks in wild ruminants where EHDV cases are differentiated from BTV cases^5–7^. Thus, the term hemorrhagic disease refers to diseases caused by EHDV and BTV, as these two viruses cause similar clinical signs and pathological findings, which are indistinguishable without laboratory diagnostic testing^8^.

There are seven EHDV and 27 BTV serotypes around the world. Three EHDV serotypes and 15 BTV serotypes have been documented in the USA^9^. The viruses are transmitted through the bite of infected midges of the genus *Culicoides*^10^. Two species of *Culicoides* are confirmed vectors of HD in the USA, *Culicoides sonorensis* and *Culicoides insignis*^11,12^. The former is considered the primary vector in North America due to its broad distribution while the latter is limited to the extreme southeastern USA but appears to be expanding^13^. However, it is suspected that additional species could be acting as vectors, contributing to augment transmission during outbreaks^9^. *Culicoides* develop in aquatic and semi-aquatic habitats, including streams, ponds, moist soils, and mud, depending on the species^14,15^. Furthermore, *C. sonorensis* preference for livestock habitat for laying eggs has been reported, as manure and highly organic moist soils are ideal for *C. sonorensis* larval development^16–18^. Climate and weather variables (e.g., temperature, humidity, wind velocity) affect *Culicoides'* life cycle and home range, thus influencing outbreak dynamics^19–24^. In northern latitudes, outbreaks occur in late summer, ending mid to late fall^25^.

Other wild ruminant species such as mule deer (*Odocoileus hemionus*), pronghorn (*Antilocapra americana*), North American elk (*Cervus canadensis*), and bighorn sheep (*Ovis canadensis*) can contract EHDV; however, WTD are particularly susceptible to severe disease associated with EHDV^26,27^. During a severe EHD outbreak in captive WTD in Missouri in 2012, the average within-herd morbidity rate was 46%, while within-herd mortality rates ranged from 3 to 84%, with an average of 42%^28^. Herd mortality in wild WTD in Missouri was estimated at up to 16%^29^; however, in locations where deer populations may not be regularly exposed to EHDV, mortality can be much higher^30,31^. Because of the potential for large die-offs during severe outbreak years, EHD remains an important viral disease of WTD in Illinois and North America^32^.

Population-level studies of HD on wild WTD would assist in the development of strategies to help manage a public resource that impacts local economies, especially because HD outbreaks often precede or coincide with hunting seasons. Despite recognizing the disease since the late 1800s in the USA and increasing reported cases in recent decades, HD's spatial and temporal distribution in wild WTD is understudied. Spatial epidemiology methods have been used to analyze infectious diseases in wildlife, including chronic wasting disease in Illinois WTD^33^, HD in WTD in Missouri^34^, and *Baylisascaris procyonis* (raccoon roundworm) in southern Ontario, Canada^35^. Analyzing large datasets using retrospective space–time permutation models helps focus surveillance efforts on high-risk areas to detect disease outbreaks^36^.

Past HD studies in the Midwest focus on region-wide trends in HD expansion, the detection and occurrence of new HD serotypes in the USA, and specific outbreak years in domestic and wild ruminants^5,6,28,37^. However, studies on HD in wild ruminants in the Midwest are still lacking^8,11^. Recent studies suggest HD outbreaks are occurring in more northern latitudes^5,38^. However, most HD data in wild WTD often report only the annual, county-level presence or absence of disease^8^ rather than numerical counts of dead deer. Thus, characterizing outbreaks in wild herds across time and space is difficult. Space and time clusters of local HD outbreaks in wild WTD populations could reveal expanding virus and vector distribution in Illinois that could be caused by changes in land use (e.g., habitat loss or agricultural development) and climate. Spatial and temporal modeling of HD outbreaks in wild WTD would highlight research gaps in the epidemiology and surveillance of HD in Illinois and identify future research directions.

BTV infection in cattle and other domestic livestock is well studied in the USA, and there is evidence of BTV seroprevalence in Illinois cattle^39^. However, evidence suggests that BTV infection in Illinois WTD may be low^40^. In contrast, the three EHDV serotypes (EHDV-1, -2, -6) known to occur in the USA have been documented in Illinois WTD^37^. The once exotic EHDV-6 was first isolated from wild and captive WTD in Indiana and Illinois in 2006, followed by Missouri, Texas, and Kansas between 2006 and 2008^37^. During fall of 2013, the first EHD outbreak in domestic ruminants was reported in Illinois. At least 18 cattle herds in northwestern Illinois were affected^41^. This outbreak coincided with the EHD outbreak affecting wild WTD in that region^41^ and followed the major outbreaks in domestic ruminants in the USA in 2012^28^. Cattle and deer have been considered important vertebrate hosts in the EHDV/BTV transmission cycle^9,42^. Furthermore, recent studies in the USA have demonstrated animal host density^43^ and biting midges preference patterns for different Cervidae and Bovidae hosts^44^ as important factors for maintaining HD viruses in captive ruminant herds. The occurrence of HD in captive herds may have consequences for subsequent infection in wild WTD populations^9^. In Illinois, cattle and deer often inhabit the same large-scale geographic areas and are often close to habitats where HD competent vectors occur. However, because of the severe impact of HD and the unknown occurrence of BT in wild WTD in Illinois, targeted surveillance efforts are needed to identify risk factors contributing to HD outbreaks in wild populations.

Currently, there are no studies on the historical occurrence of EHD or BT in wild Illinois WTD. Spatial epidemiology can identify areas with increased disease rates. Therefore, a preliminary investigation of increased HD rates to identify areas and periods with higher-than-expected HD reports will serve as a baseline for future studies. Additionally, such baselines can guide future studies to evaluate additional gaps in knowledge related to local risk factors, predominant serotypes, competent vectors, and potential animal-vector-environmental interactions favoring HD outbreaks in wild WTD populations. Therefore, our objectives are (1) determine the distribution of HD reports in wild WTD by county in Illinois and characterize the intensity and temporal intervals of HD outbreaks, (2) identify records that indicate the presence of BTV in wild Illinois WTD, and (3) evaluate geographic expansion trends of HD in Illinois using a space–time scan statistic model. To achieve the analytical components of the study objectives, we constructed a choropleth map illustrating county-level numbers of HD cases and an isopleth map to account for the areal bias associated with county boundaries. Furthermore, we used a hot-spot analysis to identify counties with significantly higher and lower numbers of HD cases. Finally, we used a space–time permutation scan that offers a time component for the identified HD clusters in addition to the spatial statistical steps.

## Results

### Descriptive analysis

**Figure 1:**
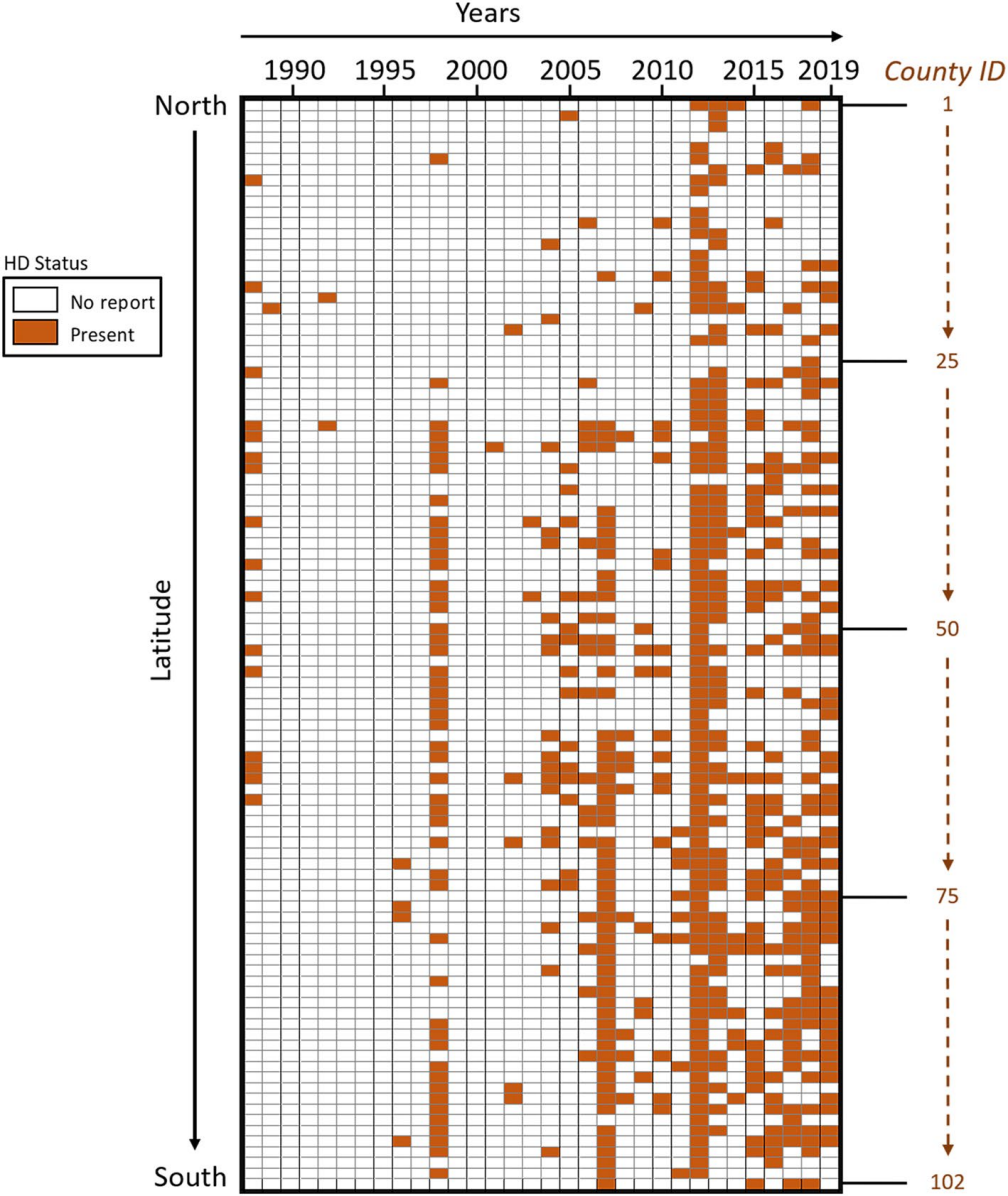
Hemorrhagic disease (HD) reports in wild white-tailed deer (*Odocoileus virginianus*) in the state of Illinois through time (1988–2019) by decreasing latitude. Individual cells in a column represent each county in the state of Illinois and are assigned a county ID 1–102; The ID number and county name are presented in Supplementary Figure S1.

**Figure 2:**
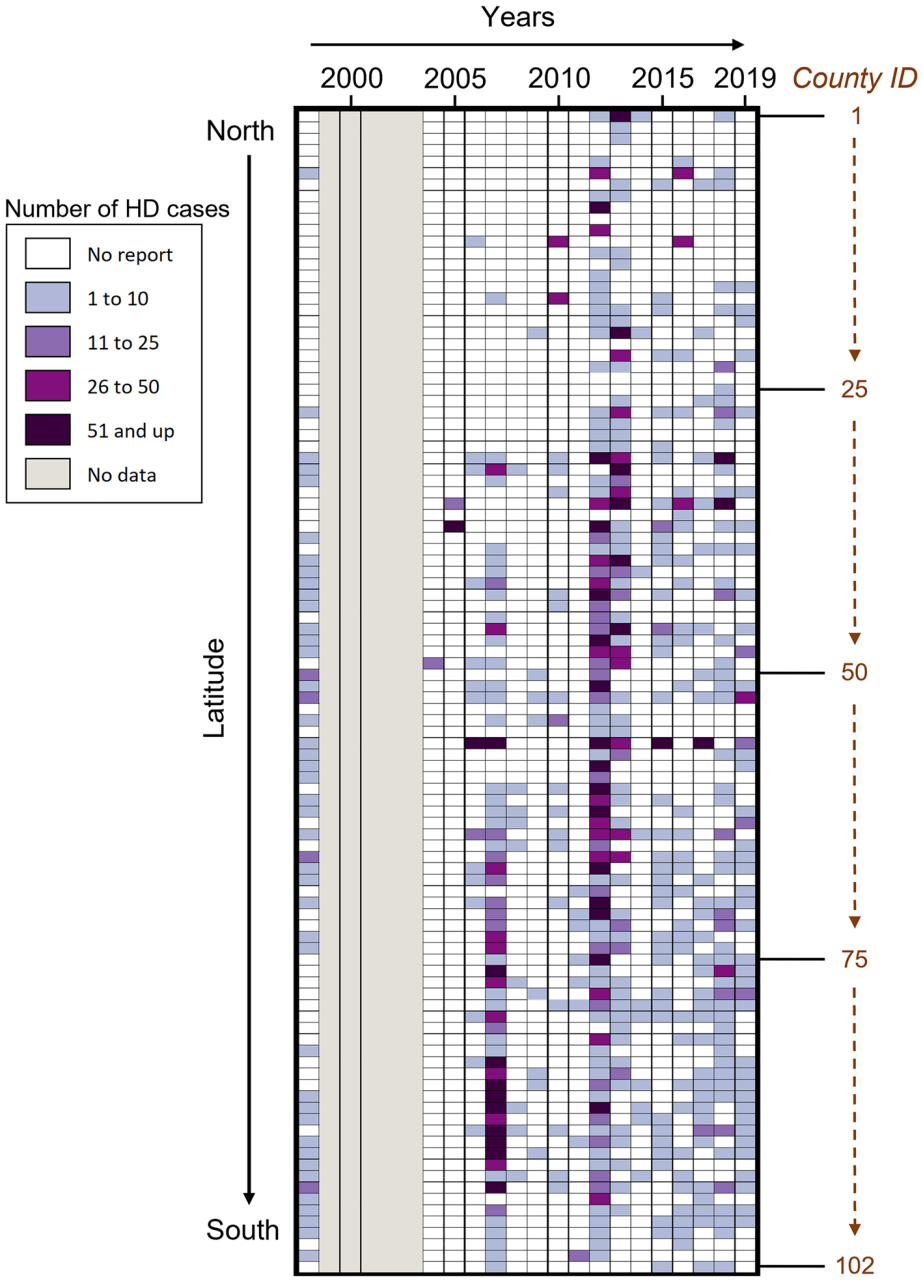
Number of hemorrhagic disease (HD) cases per county in wild white-tailed deer in Illinois by decreasing latitude from 1998 to 2019. Individual cells in a column represent each county in the state of Illinois and are assigned a county ID 1–102; The ID number and county name are given in Supplementary Figure S1.

From 1982 to 2019, 99/102 Illinois counties reported HD at least once, and three counties (Boone, DeKalb, and Kankakee) had zero reports of HD. One county (Grundy) reported presence of HD but not the number of deer with HD cases (Fig. 1). No HD cases were reported in 14 of the 38 years studied (1982–1987, 1990–1991, 1993–1995, 1997, and 1999–2000). We documented 8044 total HD deer (including reported dead deer from outbreaks and confirmed HD deer from diagnostic results). There was an average of 4.6 cases/county/year for the years that reported numerical data ((8044/102 counties)/17 years with reported cases). In 2012, 2969 cases were reported across 87 counties. Additionally, 2007 and 2013 were important outbreak years, with 1989 cases reported across 58 counties and 1227 cases reported across 63 counties, respectively. There were eight years (2005–2007, 2012–2013, 2015, and 2017–2018) in which more than 51 cases were reported within a single county (Fig. 2). Among these, the highest reported numbers were 376 cases in Lawrence County in 2007 and 359 cases in Cook County in 2012. There were 338 HD confirmed diagnostic test results for both EHD and BTV (this included all the available information from serology and virology for calendar years 2005–2019; Supplementary Table S1). From the 338 deer, 337 were tested for BTV and 254 were tested for EHDV (Supplementary Table S1). The results indicated that 4% (13 /337) deer tested positive for BTV, and 17% (58/254) tested positive for EHDV. Five deer tested positive for BTV and EHDV antibodies.

### Disease mapping

**Figure 3:**
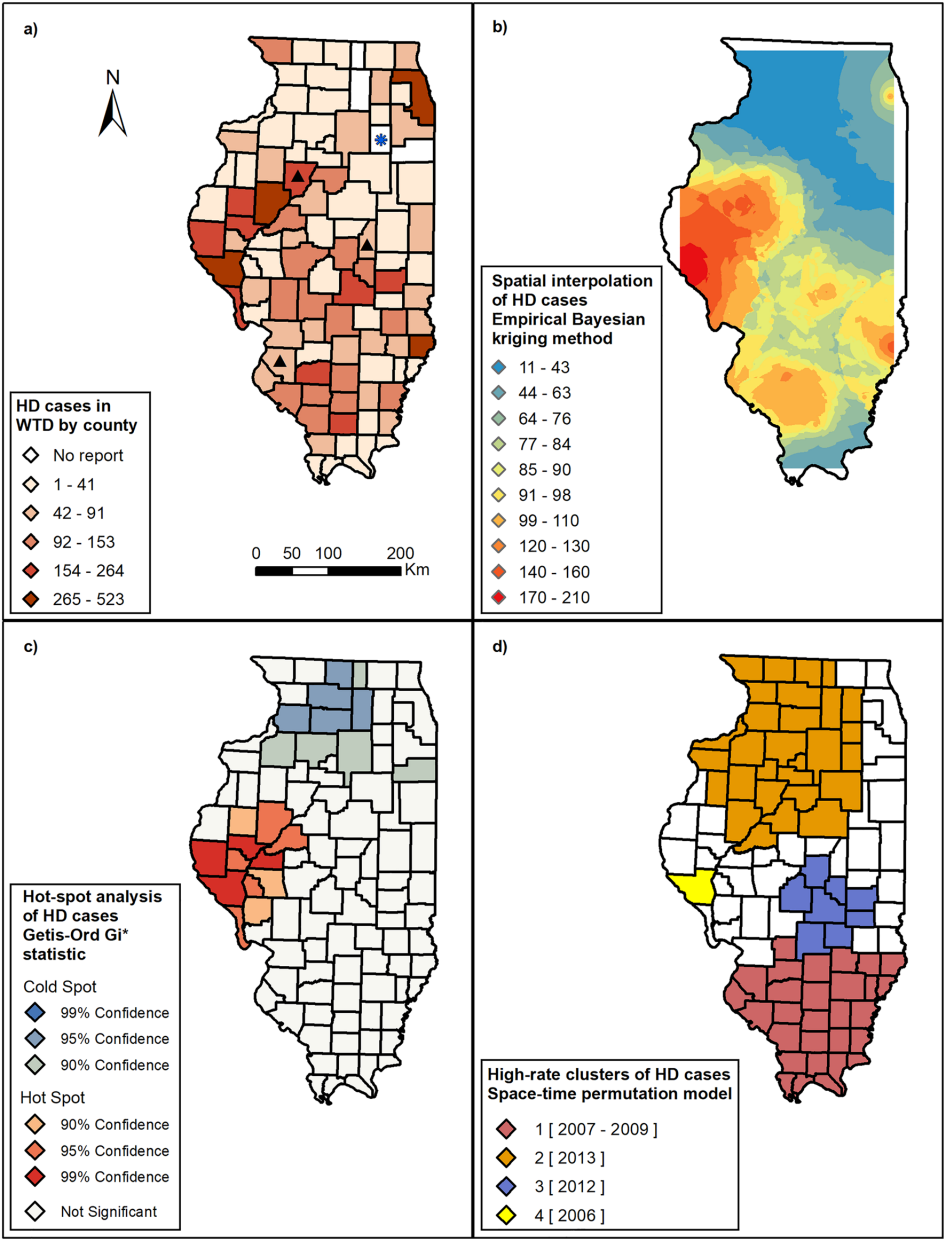
Hemorrhagic disease (HD) in wild white-tailed deer in Illinois between 2006 and 2019. (**a**) Distribution of the total number of HD cases by county in Illinois.; the asterisk indicates county where there is no numerical data, but HD has been reported as present; the triangles indicate counties in which Bluetongue was identified in wild white-tailed deer. (**b**) Isopleth map illustrating the distribution of HD cases across Illinois by using the Bayesian kriging spatial interpolation empirical method. (**c**) Hot-spot analysis of HD cases in wild white-tailed deer by using Getis-Ord Gi* statistic. (**d**) High rate clusters of HD cases identified by the space–time permutation scan statistic. Maps created using ArcGIS 10.7.1 (ESRI Inc., Redlands, CA, US).

The total number of HD cases/county for the full study period ranged from zero to 523 cases. Counties with a high number of HD cases were in west-central, southeast, and northeast Illinois (Fig. 3a).

Empirical Bayesian kriging results are illustrated in an isopleth map (Fig. 3b). Several regions with an increased number of HD cases were detected, including the west-central, northeast, and south regions of Illinois.

The hot spot analysis identified five counties with a low number of HD cases (cold spots) in north-central Illinois and nine counties with a high number of HD cases (hot spots) in east-central Illinois (Fig. 3c).

### Space–time scan statistic

The retrospective space–time scan statistic detected four high-rate clusters (Fig. 3d; Table 1). The primary cluster (C1) of 1580 cases from 29 counties was in southern Illinois, occurring 2007–2009. The second cluster (C2) of 754 cases from 29 counties was in northwestern Illinois in 2013. The third cluster (C3) of 731 cases from eight counties was in central Illinois in 2012. The fourth cluster (C4) of 103 cases was in a single county in west-central Illinois in 2006.

**Table 1:** Space–time clusters of hemorrhagic disease in wild white-tailed deer in Illinois between 2006 and 2019.

Clusters	County number	Radius (Km)	Time frame	Observed (O)	Expected (E)	O/E	Test statistic	*P*-value
C1	29	20.12 km	2007 to 2009	1580	628.95	2.51	570.50	< 0.001
C2	29	13.74 km	2013	755	219.91	3.43	415.57	< 0.001
C3	8	6.81 km	2012	732	309.90	2.36	219.22	< 0.001
C4	1	0	2006	103	10.07	10.23	147.12	< 0.001

The retrospective spatial analysis, scanning for clusters with high rates, uses a space–time permutation model with the SaTScan software; Spatial unit: centroid of a county; Temporal unit: year; Circular scanning window size of 50% of population at risk, and 50% of study period.

## Discussion

This spatial epidemiology study allows identifying high-risk areas supporting future multidisciplinary research that will help determine potential animal-vector-environmental interactions during outbreaks of HD on wild WTD populations. Furthermore, it will guide field studies to evaluate the serotypes, competent *Culicoides* spp., and host reservoirs present at local scales.

The first HD space–time cluster (C4) occurred in 2006 and affected one county (Fig. 3d), Pike County, along the Missouri border in west-central Illinois (Supplementary Figure S1). Between 2007 and 2009, HD cases were reported in 29 counties in southern Illinois, making this cluster (C1; Table 1) the biggest in size and observed cases. Similar to clusters C4 and C1, clusters C3 and C2 were identified in two consecutive years, 2012 and 2013. While these clusters show a pattern of expansion from a smaller number of the counties affected in 2012 (C3 = 8 counties in central Illinois) to 29 counties in northern Illinois affected in 2013 (C2), the radius of the clusters remained smaller than C1. The number of observed cases between 2012 and 2013 was similar but smaller than C1 in 2007–2009 (Table 1). We note that county sizes are not as variable as other states, potentially reducing some spatial biases. Interestingly, a northern expansion was first noticed during 2012–2013 outbreaks. The outbreak in Illinois in 2012 was contemporaneous with outbreaks across the USA in which 35/50 states reported cases of suspected (5 states) or confirmed (30 states) HD^8^. In 2006, four counties had their first reports of HD. However, in 2012, 18/102 counties in Illinois had a first HD report. Cluster C2, located in northern Illinois, occurred in 2013, and two more counties had their first HD report. The clusters illustrate that HD has been moving north. By 2019, 99/102 counties had reported HD cases and/or presence (Fig. 3a). These findings of northern expansion are consistent with previous reports^5^ and with the high morbidity and mortality usually reported when naïve populations are exposed to HD viruses^9^.

During the timeframe of each cluster, Illinois experienced drought in the late summer and fall months, especially in 2012 when the state experienced extreme drought conditions along with much of the US^5,28,46,47^. Although not evaluated in this study, the correlation of HD outbreaks with drought and other climatic variables (e.g., temperature, humidity, wind) has been a focus of recent studies^5,21,23,48,49^. Drought has been suggested to contribute to HD outbreaks^21,28^. Warm and shallow water with freshly exposed mud is ideal for midge reproduction, especially *C. sonorensis*^50^. The congregation of animals due to limited water sources may contribute to increased exposure to *Culicoides*^50,51^, mud disturbance, and fecal matter deposition, increasing habitat suitability for propagation and reproduction of *Culicoides* vectors^9^. The role that drought and other climatic factors may play in vector dynamics, virogenesis, and HD outbreak severity could be important considerations in anticipated changes in climate and hydrology^52,53^. Although the clusters identified in this study were associated with years where drought was reported in Illinois, not all HD outbreaks in the USA are related to drought conditions, as demonstrated in the eastern USA in 2017^54^. Therefore, along with climate impacts on vector activity, other biotic and abiotic factors may impact HD transmission and outbreak severity. Landscape characteristics^55^, soil chemistry^17,56^, land-use practices, and environmental conditions associated with livestock^18,57–60^ (e.g., overflow from stock tanks, livestock wastewater lagoons, soil disturbance along riparian areas or stock ponds) may also impact vector abundance and vector-host interactions. A better understanding of how these and other factors affect the disease system could inform risk communication and management decisions to guide hunter harvest in outbreak areas and sustain healthy populations of wild deer in severe outbreaks.

Many HD positive counties are located along the Illinois River and are included in three of the four clusters (C2, C3, and C4) identified by the space–time analysis (Fig. 3d). Pike County was identified as a cluster (C4) in 2006 and is part of the nine counties identified as hot-spots for HD cases in Illinois. This county reported HD in nine of the 38 years of data. Pike County is a mix of forested and agricultural land between the Mississippi and Illinois Rivers (Supplementary Video 1). This county consistently reports some of the highest annual deer harvest numbers in the state^61^, which may be due to higher deer densities, increased hunting participation, and the perceived “trophy quality” of Pike County WTD. These same factors may also contribute to higher reporting of HD because the public may be more invested in this resource, thus contributing to increased surveillance and sampling efforts. Higher deer populations may also contribute to the congregation of reservoir ungulate hosts, facilitating exposure to infected *Culicoides*^62^. Because of higher deer populations^61^ and possibly because of the availability of vector habitat (e.g., wetlands, stock ponds, and exposed riparian areas), Pike County and counties with similar physiographic characteristics along the Illinois River may be more prone to HD outbreaks than other parts of the state. The study of animal movement around water sources and higher coverage of water bodies have not been evaluated in association with HD in Illinois. While this study did not evaluate river corridors and their relation to HD outbreaks, we recognize that defining the role of animal movement around water sources and the link of the amount and type of water bodies by county on disease spread may help determine inter-related variables affecting HD dynamics. Incorporating these variables in future HD epidemiological models may contribute to understanding the changing patterns and distribution of HD in Illinois.

Previous studies have identified multiple factors affecting host-vector interactions and vector distribution and abundance. High animal densities in captive populations—independently if animals are kept in a fenced enclosure, open pasture, or free-roaming in natural preserves—have been identified as significant contributors to HD presence compared to wild populations in the same geographic area^43^. Additionally, the preference patterns of different *Culicoides* for different ungulate species have also been suggested as a contributing factor in HD dynamics between ruminant hosts^44^. Facilities with exotic ungulates, although not developing the disease, could contribute to local EHDV/BTV transmission^43^. The high density of animals can impact landscape characteristics and contribute to higher vector density^63,64^. Other factors such as climate and weather can affect vector ecology. Although unclear which physiographic features could be responsible for the increase in reported HD cases during the outbreaks in Illinois, identifying the hot-spot/cold-spots in specific areas and during a period can help guide future surveillance and management efforts at the county level. Further investigation of climatic features, *Culicoides* spp., artificial changes to the environment (e.g., urban development and agricultural practices), animal movement between farms, and other risk factors in hot-spot areas can help identify potential predictors for future outbreaks in specific areas in Illinois. Recognizing the specific risk factors by area will help develop more specific management plans to control disease dissemination in domestic ruminants, thus protecting other domestic and wild ruminant populations. While we did not evaluate geographical factors affecting HD reporting, deer that die from HD may be more likely to be detected in areas closer to urban settings than those in rural settings where they may go undetected. Host distribution differs between urban and rural areas, and therefore, die-offs are more likely to be reported in areas with deer habitat and greater host densities. Outdoor activities such as farming, hiking, or hunting may increase HD reports.

Hemorrhagic disease data in Illinois are obtained through diagnostic submissions, the Southeastern Wildlife Cooperative Disease Study (SCWDS) annual HD questionnaire, and available harvested deer samples tested for HD outside of the outbreak season. Historical data pertaining specifically to HD in Illinois wild WTD was difficult to obtain. The earliest available reports of HD in Illinois date to 1982. Since 1982, SCWDS has conducted a nationwide HD survey of state wildlife management agencies. The goal of the survey is to monitor county-level reports of suspected and confirmed HD in wild WTD and other wild ruminants. However, the survey does not consistently capture the number of deer affected in each county or state, but it may capture county-level presence. The Illinois Department of Natural Resources (IDNR) provided annual HD reports beginning in 1998, which included county-level counts of reported dead deer. Beginning in 2013, available IDNR data included more detailed information regarding case reports (e.g., location, date, sex, and age), although this is not always included in the records. We recognize the challenges and limitations to conducting HD surveillance during outbreaks, which include: (1) the time of year (late summer to early fall) when decomposition may accelerate due to high temperatures, (2) location of carcasses (commonly found in water), and (3) limited availability of field staff for sampling a high number of dead deer during large-scale outbreaks. All these factors make it extremely difficult to collect the necessary tissue samples for HD diagnostics in a timely manner (within 24–48 h of a death to avoid decomposition), impacting sampling efforts over the years.

There is insufficient information to know how much the sampling effort varied over the years. While recognizing the challenges and difficulty in gathering this information for every HD dead animal during an outbreak, variations in the data make complex data analyses challenging. To advance the quantification and analysis of risk for an outbreak of HD in wild WTD and to permit the development of better predictive models for HD outbreaks, officials should standardize the data collected from HD reports and field visits to include: date of reported case, county, TRS (township, range, and section number), geographic coordinates, number of dead deer reported, age (fawn, yearling, or adult), sex, and sample ID (if tissues are submitted to a diagnostic laboratory).

Using available Illinois historical HD data, we identified diagnostic results for wild WTD in Piatt County, Illinois, with antibodies for BTV. Eleven deer samples from 2005 to 2006 tested positive for antibodies for BTV. Of those, five tested positive for antibodies for both EHDV and BTV. Cross-reaction is known to occur when using the AGID test to identify BTV and EHDV^65^. However, because six of those deer samples tested positive for BTV antibodies and negative for EHDV antibodies, cross-reaction may not have occurred in these cases. The deer that tested positive for antibodies against BTV were all harvested from the same area in Piatt County. These Piatt County deer and two additional deer found positive for BTV in Peoria and St. Clair counties from a previous study^40^ demonstrate that BTV exposure has likely occurred in wild WTD in different regions of central Illinois. However, available diagnostic results of wild WTD statewide suggest that EHDV dominates HD mortality in Illinois deer. None of the deer tissue samples submitted during the outbreak months tested positive for BTV. This agrees with findings in domestic ruminants during the 2013 outbreak, where four northern Illinois counties (Kane, Jo Daviess, Henry, and Stephenson) reported EHDV infection in cattle, but no BTV cases^41^.Thus, while we know that BTV is present in Illinois as evidenced by seropositivity in Illinois cattle^39^ and that deer become infected with BTV, we do not know its distribution or the extent to which wild WTD are infected and affected by this virus. Domestic cattle are considered an important host for BTV and EHDV^66^. However, the role of wild WTD as an important host has been associated only with EHDV^9^. However, cattle may not be the only farmed ruminant that plays a crucial role as a host for BTV/EHDV. For example, a recent study identifies 44% of mortality cases in farmed WTD in Florida between 2012 and 2020 associated with BTV, EHDV, or co-infection of BTV/EHDV^67^. This study demonstrates the impact of these viral diseases in captive cervids and highlights the importance of continued surveillance and using farmed ruminants as sentinels for HD in the surrounding wild ruminant populations^67^.

Nevertheless, our results corroborate that, despite detecting some cases with positive EHDV and BTV results, EHDV was the orbivirus driving the HD cases in wild Illinois WTD (Supplementary Table S1). Previous studies have identified that increased temperatures can affect vector survival and competence for different serotypes. For instance, temperatures between 27 and 30 °C increase vector competence for EHDV (serotype 1); however, these temperatures interfere with vector competence for BTV (serotypes 10 and 16)^23^. Although we did not evaluate the effect of temperature on the HD outbreaks in Illinois, we recognize the importance of expanding HD surveillance to identify the association of environmental variables with distribution and abundance of competent *Culicoides*. Expanded surveillance will help detect the incursion of new virus strains to Illinois and to North America, while establishing the most common serotypes in Illinois. Only through surveillance can a complete picture of the variables associated with the EHDV/BTV triad (vector-host-environment) be developed and used to assess management programs designed to protect wild and domestic ruminants.

We demonstrated that HD cases in Illinois cluster in space and time, suggesting that we are dealing with the localized transmission of the virus in a period. Additionally, we found an expansion of HD to areas of the state that had previously not reported cases. However, we did not evaluate ecological factors (e.g., changes in deer behavior and population dynamics and *Culicoides* population dynamics) and anthropogenic factors (e.g., landscape changes, introduction or translocation of wildlife and livestock) that may impact severe HD outbreaks. Notably, cases in earlier years were reported from central and southern Illinois, and in later years, they were also reported in northern Illinois (Fig. 1). Regional patterns in HD outbreaks have historically fallen into one of two categories: enzootic (occurring every 2 to 3 years) or epizootic (occurring every 8 to 10 years)^25,68^. Figure 1 and a time series plot of the number of counties reporting HD occurrence between 1988 and 2019 (Supplementary Fig. S2a) revealed three peaks of HD in 1998, 2007, and 2012. The peaks also demonstrate an increase in counties with a HD report. Visually inspecting Fig. 2, we observe a cyclical pattern with a high number of HD cases reported in 2007 and 2012–2013. This pattern is also observed in a time series plot (Supplementary Fig. S2b). This observation was confirmed by the scan statistical analysis where three large space–time clusters of the number of cases were detected in the south, north, and central regions of Illinois during these periods (Fig. 3d). Our study identified BTV and EHDV exposure in deer through general disease surveillance outside typical HD outbreak periods and highlights a potential gap. By limiting surveillance to outbreaks, we do not detect areas where BTV and/or EHDV infected deer survived. While HD outbreaks may be seasonal, surveillance efforts, particularly serological surveys, should be conducted year-round.

Although we did not evaluate environmental factors and the biological vectors influencing HD transmission in Illinois, we recognize the importance of climate change on emerging and re-emerging viral diseases at a local and global scale^9,69^. For instance, the identification of EHDV-6 in Indiana and Illinois in 2006, Missouri in 2007, Kansas and Texas in 2008, and Michigan in 2009 were suggested as a result of overwintering maintenance and, therefore, expansion of EHDV in the USA^70^. However, there is no evidence of the virus overwintering in northern areas; therefore, this remains to be evaluated. During the 2006 EHDV outbreak in Israel, winds were considered a major contributing factor to HD spread^71^. Additionally, the maintenance of BTV in a farm in temperate regions in northern California was associated with prolonged survival of *C. sonorensis*, thus contributing to interseason maintenance^72^. Therefore, there are local and global scale indicators of environmental factors affecting HD transmission that should not be overlooked. Moreover, phylogenetic analysis of virus strains may help identify the movement of virus populations^70^ and the genetic and spatial distance of virus strains identified during outbreaks. Such findings may point to other variables contributing to virus transmission, dissemination, vector competence and maintenance. HD surveillance on farmed ruminants may serve as a sentinel strategy to identify risk factors for disease transmission in captive and wild populations^67^. Livestock and captive cervid facilities may be valuable sentinels for HD surveillance because it is possible to identify the recently infected (viremic) animals necessary for the infection of biological vectors^43,67^. Evaluation of native *Culicoides* species near captive facilities may help detect shifts in HD occurrence and the potential for maintenance of HD viruses outside the known vector home range^9^.

In regions like Illinois, HD outbreaks may result in high mortality and impact local economies and tourism, especially the outdoor recreation industry. However, HD outbreaks also affect the agricultural industry and captive cervid herds. Recognizing the importance of HD, IDNR collects detailed information when investigating reports of dead deer. However, it is not always possible to collect geographic coordinates for each dead deer. Nevertheless, geographic coordinates are critical for fine-scale analyses of outbreaks and future studies on vector, landscape, and natural and agricultural conditions that may play a role in HD outbreaks. When the collection of tissue samples is not possible (e.g., poor post-mortem condition of the carcass) or field visits are not feasible (e.g., too many reports or limited staff), location data should be collected. TRS location data provides the next best option and offers an opportunity to evaluate problem areas where repeated outbreaks occur. Additionally, TRS-level data can assist with predictive models of disease expansion while accounting for variables like wetlands, forests, soils, and proximity to livestock facilities. Predictive models can help wildlife agencies prepare resources for outbreak response, inform the public of expected outcomes, and help inform management decisions (e.g., adjusting harvest quotas) to sustain healthy populations of deer in areas of severe HD outbreaks.

## Methods

### Study area

Illinois is a state in the Midwest region of the USA and is made up of 102 counties (Supplementary Fig. S1). It is roughly 150,000 km^2^, ranges in latitudes from 42.5° N to 37° N, and is approximately 630 km long from north to south. Illinois’ climate is temperate and includes four seasons. The average annual precipitation ranges from ~ 1200 mm in the south and ~ 812 mm in the north. Illinois is predominantly agricultural land mixed with moderately to highly dense urban areas and some deciduous and deciduous-mixed forest land, prairie, and wetlands.

### Types of HD data

Categorical data on the presence/absence of HD were obtained from SCWDS for the years 1982–2006, 2009, and 2019 and from the United States Geologic Survey National Wildlife Health Center WHISPers database (WHISPers)^73,74^ for 2004. For 2005, Illinois Department of Natural Resources (IDNR) provided estimates of dead deer, and those records were considered as categorical data. IDNR also provided reports with numerical data (including counts of suspected and confirmed HD dead deer found during an acute mortality event) for 1998 and 2006–2019. Confirmed HD cases were verified from submitted tissue samples (e.g., spleen, liver, kidney, lymph node, and tongue) analyzed by the Veterinary Diagnostic Laboratory at the University of Illinois Urbana-Champaign (VDL), Newport Labs, and SCWDS using real-time reverse transcription polymerase chain reaction (rRT-PCR) according to standard laboratory protocols. In addition, the Wildlife Veterinary Epidemiology Laboratory of the Illinois Natural History Survey provided numerical data from 2004, 2006, and 2007 that included HD diagnostic results from harvested deer (serum samples) from Piatt County, Illinois. For this data source, the VDL used the agar gel immunodiffusion assay (AGID) diagnostic test (Veterinary Diagnostic Technology Inc., Wheatridge, Colorado, USA), and all the deer that tested positive for BTV and/or EHDV were added to the data. The VDL analyzed serum samples from Piatt County’s harvested deer using the agar gel immunodiffusion assay (AGID) diagnostic (Veterinary Diagnostic Technology Inc., Wheatridge, Colorado, USA). The United States Department of Agriculture Animal and Plant Health Inspection Service—Wildlife Services (WS) provided diagnostic data for BTV in wild WTD for the years 2010–2015. Serum samples were analyzed using the 80% plaque reduction neutralization test^40^.

### HD data collection in Illinois

Currently, in Illinois, HD surveillance is achieved through IDNR investigation of public reports of dead deer. Deer reported as having died from HD met at least one of the three mortality criteria suggesting acute HD: (1) high and acute mortality events during late summer and early fall, often near a water source (e.g., river, creek, pond), (2) HD diagnosis from necropsy findings, or (3) dead deer with laboratory confirmation of hemorrhagic disease (EHD or BT)^32^. IDNR staff record information from all HD reports including the number of dead deer and the location of the outbreak (at least the county level). Field investigations are conducted whenever possible but are limited by staff availability and the condition of the carcasses reported. Priority is given to counties in which HD has yet to be confirmed in a given year. Once HD is confirmed in a county, it is generally not revisited for laboratory confirmation of other dead deer during the same season. Circumstances that affect the collection of useful diagnostic samples include temperature, location of the carcass, and age of the carcass. The level of tissue decomposition increases with time, environmental temperature, humidity (in air and from carcasses found in water)^75^. The number of days post-mortem before the carcass can be examined by biologists affects the quality of a sample available for diagnostic testing. Thus, IDNR staff collect spleen and/or lung samples from fresh deer carcasses (usually within ~ 48 h of death) for laboratory confirmation (IDNR personal communications). Reported HD cases are logged by IDNR and used in this study.

### Descriptive analysis

We used numerical and categorical “presence” of HD cases in a county. We excluded information from captive deer. We converted the numerical count data to “presence” and merged it with the categorical data from SCWDS (1982–2006), WHISPers (2004), and IDNR (2005) to produce an HD “presence” dataset. We screened for duplicate records across sources and removed duplicates.

Although HD data collection in Illinois began in 1982, the first reported cases of HD did not occur until 1988; therefore, we included only the years 1988 to 2019 in our analysis of HD presence.

We organized the Illinois counties by latitude from north to south using the county centroid and provided a visual representation of HD presence (Fig. 1) and numerical data (Fig. 2) for HD cases in wild WTD.

### Disease mapping

Disease mapping, kriging, and the local hot spot analysis were performed using Spatial Statistics and Geostatistical Analyst Tools in ArcGIS 10.7.1 (Environmental Systems Research Institute, Inc., Redlands, CA, USA).

All reported HD cases during the study period were geocoded and spatially joined to the county level. The total number of HD cases by county was illustrated in ArcGIS 10.7.1 in a choropleth map.

Spatial interpolation of the county-level total number of reported HD cases was done using the Empirical Bayesian kriging method. To measure the spatial dependence of HD cases across Illinois counties, kriging uses a semivariogram, a function representing the distance and direction of two locations (e.g., counties). The Empirical Bayesian kriging accounts for the error created by estimating the semivariogram parameters by building several semivariograms instead of one and uses a restricted maximum likelihood estimation^76,77^.

Hot spot analysis of county-level HD cases was conducted using the Getis-Ord Gi* method^78^. Each county was represented by a polygon, its centroid, and its total number of reported HD cases for this analysis. The null hypothesis assumes complete spatial randomness of counties with high or low reported HD cases. The statistic provides a Z-score and *p*-value, which are associated with the standard normal distribution. High (positive) or low (negative) Z-score, associated with small *p*-values, are observed in the tails of the normal distribution and signify a statistically significant pattern. A significant (*p* ≤ 0.05) hotspot area is indicated by a high positive Z-score (≥ + 1.96) where counties with a high number of HD cases are surrounded by counties with a high number of HD cases. Likewise, a statistically significant cold spot (*p* ≤ 0.05) area is signified by a high negative Z-score (≤−1.96) and is detected if counties with a low number of HD cases are surrounded by counties with a low number of HD cases. To adjust for zoning (e.g., two counties might have the same number of cases but the county shapes and boundaries differ) and edge effects (e.g., counties at the periphery of the study area have neighboring areas that are not included in the study region), the “zone of indifference” conceptualization parameter was used.

The spatial interpolation and hot spot analysis results of county-level HD cases were illustrated in ArcGIS 10.7.1. The space–time permutation model requires only case data without a need for additional population-at-risk data. The model identifies disease clusters by comparing the observed number of cases in a specific area during a period to the number of expected cases that would occur if the spatial and temporal locations of all cases were independent of each other. It does not make prior assumptions on the size, location, or period of the outbreaks. It accounts for purely spatial and temporal variations in disease rates. For example, clusters will not be identified if during a specific period all regions have an increased number of cases than normal. However, clusters will be identified if cases increased in a specific region during a specific period and all the other areas have normal case numbers.

### Space–time scan statistic

Retrospective space–time analysis scanning for clusters with higher than expected HD cases using the space–time permutation model^36^ was conducted in the SaTScan software version 9.6^79^. The outcome variable was represented by the number of HD cases reported in a county in a year. The smallest spatial unit was signified by the geographical center (i.e., centroid) of a county, and the smallest time unit was signified by the year of the report. Cartesian latitude and longitude coordinates for each county’s centroid were calculated in ArcGIS 10.7.1.

The space–time scan statistic uses a cylindrical scan window with a circular spatial base and height corresponding to time^80^. A scanning window containing up to 50% of the study area and up to 50% of the study period was used. The scanning window of variable radii gradually moves through time and space, evaluating the number of HD cases inside the scanning window comparing it to the number of cases outside the window. A primary cluster with the highest significant (*p* ≤ 0.05) test statistic after a Monte Carlo hypothesis testing with 999 replications is identified. Secondary clusters are reported if they are significant and do not overlap with the primary cluster.

All significant primary and secondary space–time HD high-rate clusters were mapped in ArcGIS 10.7.1 (ESRI Inc., Redlands, CA, US) at the county level.

## Supplementary Information

Supplementary Information 1.

Supplementary Information 2.

Supplementary Video 1.

## Data Availability

Data supporting this study are available in the Supplementary Dataset.
