# Supplementary material for: Spatial epidemiology of hemorrhagic disease in Illinois wild white-tailed deer
Source: Sci Rep. 2022 Apr 27;12:6888. doi: 10.1038/s41598-022-10694-y (PMC9046210; doi:10.1038/s41598-022-10694-y)
Supplement: Supplementary file 2 — Supplementary Information 2. [file 41598_2022_10694_MOESM2_ESM.pdf]

Spatial epidemiology of hemorrhagic disease in Illinois wild white-tailed deer

*Sheena J. Dorak[1], Csaba Varga[2], Mark G. Ruder[3], Peg Gronemeyer[1], Nelda A. Rivera[1], Douglas R. Dufford[4], Daniel J. Skinner[4], Alfred L. Roca[5], Jan Novakofski[1,5], *Nohra E. Mateus-Pinilla[1,2,5]

[1]Illinois Natural History Survey – Prairie Research Institute, University of Illinois Urbana-Champaign, 1816 S. Oak Street, Champaign, IL 61820, USA.

[2]Department of Pathobiology, University of Illinois Urbana-Champaign, 2001 South Lincoln Avenue, Urbana, IL 61802, USA.

[3]Southeastern Cooperative Wildlife Disease Study, College of Veterinary Medicine, University of Georgia, Athens, GA, 30602, USA.

[4]Illinois Department of Natural Resources, One Natural Resources Way, Springfield, IL, 62702, USA.

[5]Department of Animal Sciences, University of Illinois Urbana-Champaign, 1207 West Gregory Drive, Urbana, IL 61801, USA.

Correspondence and requests for materials should be addressed to N.E.M.P. (email: nohram@illinois.edu) or S.J.D (email: sjdorak@illinois.edu).

Supplementary Information

Supplementary Table S1. Hemorrhagic disease serology and virology diagnostic results in Illinois (2005-2019). Summary of laboratory results confirming EHDV and BTV in wild white-tailed deer in Illinois. Includes results for deer tested for BTV and/or EHDV. It also includes deer from harvested deer and from the HD outbreaks if tested.

Year	Total deer tested	Deer tested for BTV	Positive for BTV	% BTV positive	Deer Tested for EHDV	Positive for EHDV	% EHDV positive
2005[a]	45	44	9	20%	45	9	20%
2006[a]	132	132	2	2%	132	4	3%
2007[a]	2	2	0	0%	2	2	100%
2008[b]	1	1	0	0%	1	0	0%
2009[b]	1	1	0	0%	1	0	0%
2011[c]	3	3	0	0%	0	0	.
2012[b,c]	4	4	0	0%	4	3	75%
2013[b,c]	63	63	0	0%	10	8	80%
2014[b,c]	8	8	0	0%	1	0	0%
2015[b,c]	32	32	2	6%	11	4	36%
2016[b]	7	7	0	0%	7	4	57%
2017[b]	4	4	0	0%	4	0	0%
2018[b]	18	18	0	0%	18	13	72%
2019[b]	18	18	0	0%	18	11	61%
**Total**	**338.00**	**337.00**	**13.00**	**4%**	**254**	**58**	**17%**

[a]Serum samples of harvested deer in Piatt County, Illinois analyzed using agar gel immunodiffusion assay (AGID) diagnostic test.

[b]Tissue samples (e.g., spleen, liver, kidney) of suspected HD dead deer in Illinois analyzed using real-time reverse transcription polymerase chain reaction rRT-PCR diagnostic test.

[c]Serum samples tested using 80% plaque reduction neutralization test.

Supplementary Figure S1. Illinois counties. The state of Illinois with County ID, corresponding county names, and neighboring states in the USA. Map created using ArcGIS 10.8 (ESRI Inc., Redlands, CA, US).

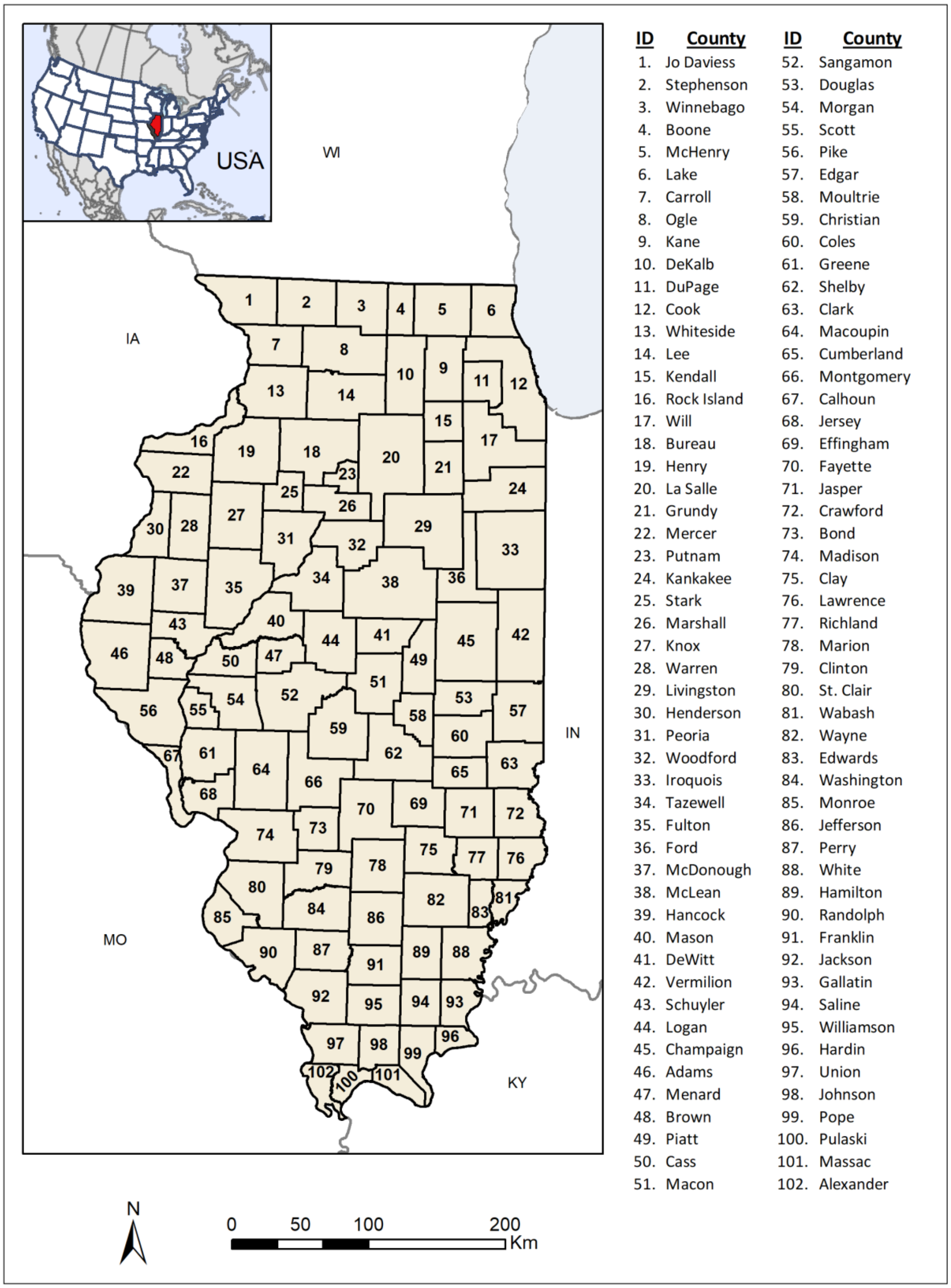

Supplementary Figure S2. Time series plots of hemorrhagic disease (HD) in the state of Illinois, USA. (a) Number of counties reporting HD occurrence (presence) from 1988 to 2019. **(**b) Number of HD cases from 1998 to 2019 (the number of cases was not reported from 1999 to 2003).

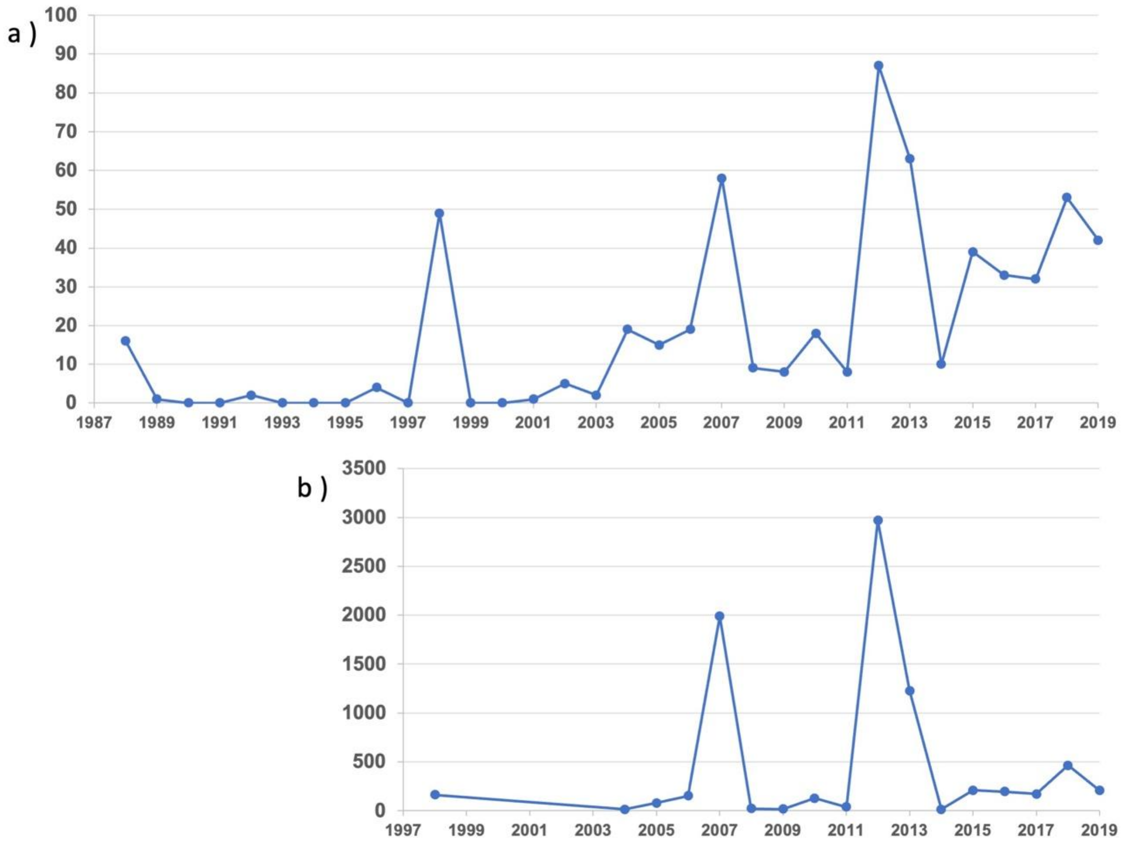

Supplementary Video 1. Hemorrhagic disease in Illinois. Animation of the number of cases in each county by year (1998 to 2019). Created by William M. Brown using ArcGIS 10.6 (ESRI Inc., Redlands, CA, US).
